# The role of interventional radiology for pediatric blunt renal trauma

**DOI:** 10.1186/s13052-015-0181-z

**Published:** 2015-10-15

**Authors:** Wei-Ching Lin, Chien-Heng Lin

**Affiliations:** Department of Radiology, China Medical University Hospital, Taichung, Taiwan; Depatment of Biomedical Imaging and Radiological Science, College of Health Care, China Medical University, Taichung, Taiwan; Division of Pediatric Pulmonology, Children’s Hospital of China Medical University, No. 2, Yuh-Der Road, Taichung, Taiwan R.O.C

**Keywords:** Interventional radiology, Renal trauma, Children, Extravasation

## Abstract

**Background:**

This study aimed to appraise the role of interventional radiology in children with blunt renal trauma.

**Methods:**

The clinical data, injury severity score, days of hospital stay, outcomes and complications of pediatric renal trauma were recorded and evaluated. The two groups: the transcatheter arterial embolization (TAE) group and the non-TAE group were compared for clinical features and laboratory data.

**Results:**

Eighteen pediatric patients (12 boys, 6 girls with average age 12.4 ± 4.7 years) with blunt renal injury were included in the study. Six patients underwent angiography because of contrast medium extravasations in the kidney found on computed tomography of which four subsequently underwent a TAE. The clinical features and laboratory data of patients in the TAE and non-TAE groups were not significantly different. All patients were managed successfully by conservative treatment without complications except one in the non-TAE group who required nephrectomy due to renal arterial hypertension directly related to trauma. Both groups had relatively good results and all patients had normal renal function at follow-up.

**Conclusion:**

TAE is an alternative therapeutic modality for blunt renal injury in children who have contrast medium extravasations in the kidney on angiography.

## Introduction

The kidney is the most common organ affected by pediatric blunt abdominal trauma because it is more mobile in children compared to adults, less perirenal fat and less protected by the ribs due to lower position in the abdomen [1–4]. The management of pediatric blunt renal trauma is based on the clinical status or severity of injury. The majority of blunt renal injuries are low-grade contusions that require no active therapy, and prompt exploration or nephrectomy is indicated only in hemodynamically unstable children refractory to blood transfusion or in case of suspected renal pedicle injury [5–9].

In adults, selective percutaneous transcatheter arterial embolization (TAE) is often performed by an interventional radiologist if active bleeding signs, a pseudoaneurysm or an arteriovenous fistula are detected on computerized tomography (CT) scan [10], thereby preserving maximal renal function [11, 12].

Despite reports of the success of TAE in adults with blunt renal injuries, experience with this technique to control ongoing bleeding from blunt renal injuries in children is limited and there is no consensus on the indications of its use in children [13]. The purpose of this study was to evaluate the role of interventional radiology in children with blunt renal trauma.

## Methods

This study was approved by the hospital institutional review board and the requirement of informed consent was waved for this retrospective research.

An analysis of pediatric patients (age ≤18 years) with blunt renal injuries admitted to the hospital between February 2005 and August 2010 was performed. Medical records of the patients were reviewed including laboratory data, site of injury, grade of renal injury, injury severity score (ISS), use of TAE, days of hospital stay, transfusion requirements and complications.

All injuries were identified by CT scan (LightSpeed 16, GE Healthcare) and graded using the Organ Injury Scaling Committee Guidelines on a scale of 1 to 5 according to the CT findings [5]. The dynamic intravenous contrast-enhanced abdominal CT is included both arterial and venous phases.

In our hospital, the initial management of pediatric blunt renal trauma patients is according to the Advanced Trauma Life Support (ATLS) guidelines. Fluid resuscitation and blood transfusion is delivered for those hemodynamically unstable patients and followed by focused abdominal sonography for trauma (FAST). Patients who remain hemodynamically unstable after fluid resuscitation and blood transfusion and with free fluid detected on FAST undergo laparotomy for the possibility of co-comitant intra-abdominal organs injury. The hemodynamically stable patients present with hematuria, flank ecchymosis, lumbar, vertebral or transverse process fractures, or fracture of the lower ribs, they undergo dynamic intravenous contrast-enhanced abdominal CT. Stable patients is defined as patients who have neither tachycardia nor hypotension, whether receiving fluid resuscitation or not initially.

If CT shows renal trauma with vascular signs including contrast medium extravasation, pseudoaneurysm, and arteriovenous fistula, an emergency angiography is performed. If angiography reveals persistent vascular signs of the kidney, superselective TAE is performed with or without a microcatheter, and either steel coils or gelatin particles or both.

Several variables were compared between the non-TAE group and the TAE group, including demographic data, laboratory data, injury scores, ISS, blood transfusion amount, and hospital stay. The possible procedure-related complications, including puncture site hematoma, re-bleeding, infection, infarction, or abscess formation were identified and recorded.

Continuous variables were expressed as mean ± standard deviation, and compared by Mann–Whitney U-test. Fisher’s exact test was used to compare the categorized data. The SPSS package (SPSS Inc version 11, Chicago, IL, USA) was used for analyses, and *p* values less than 0.05 were considered statistically significant to reject the null hypothesis.

## Results

Eighteen patients (12 boys, and 6 girls) were included and fit criteria for retrospective group analysis in the study, and their detailed characteristics are summarized in Table 1. The average age was 13.4 ± 4.7 years, with the median age being 16 years (range 2–17). The most common mechanisms of injury were traffic accident (83 %), followed by violence (11 %), and fall (5 %). Fifteen patients had high grade renal injury (≥grade 3), and the average ISS was 16.9 ± 8.1 (range 4–29). The average length of hospital stay was 10.1 ± 10.3 days. The right kidney was injured in 13 patients and the left in 5 patients. Among three patients with grade 5 injury, two (case 7 and case 17) had main renal artery injuries, while others had completely shattered kidneys with intraparenchymal vascular injuries.

**Table 1:** Characteristics of the 18 patients

Patient’s number	Sex/Age	Grade	ISS	Injured kidney	Cause of injury	Hematuria	Angiography/TAE	BT in 24 h (u)	Hospital stay (days)	Complication
1	M/16	4	16	R	T/A	N	Y/Y	8	5	
2	M/15	3	9	L	T/A	Y	Y/Y	0	8	
3	M/15	3	29	L	Violence	Y	Y/Y	4	14	
4	M/14	5	25	R	T/A	Y	Y/Y	4	17	
5	M/16	4	22	R	T/A	Y	Y/N	4	6	
6	M/17	4	16	R	T/A	Y	Y/N	4	5	
7^a^	M/7	5	25	L	T/A	Y	N/N	6	11	Splenic hemorrhage
8	M/14	4	29	R	T/A	Y	N/N	0	41	
9	F/2	2	9	R	T/A	Y	N/N	1	5	
10	F/17	3	22	R	T/A	Y	N/N	0	11	
11	F/16	3	9	R	T/A	Y	N/N	4	5	
12	F/7	3	9	R	Violence	N	N/N	0	4	
13	F/16	1	22	R	T/A	Y	N/N	4	9	
14	F/12	2	4	R	Fall down	N	N/N	0	2	
15	M/17	3	27	R	T/A	Y	N/N	10	29	
16	M/17	4	16	L	T/A	Y	N/N	0	1	
17	M/16	5	25	L	T/A	Y	N/N	2	7	
18	M/17	3	10	R	T/A	Y	N/N	0	2	

^a^This patient underwent surgery, *M* male, *F* female, *R* right, *L* left, *T/A* traffic accident, *Y* yes, *N* no, *ISS* injury severity score, *TAE* transcatheter angiographic embolization, *BT* blood transfusion

**Fig. 1:**
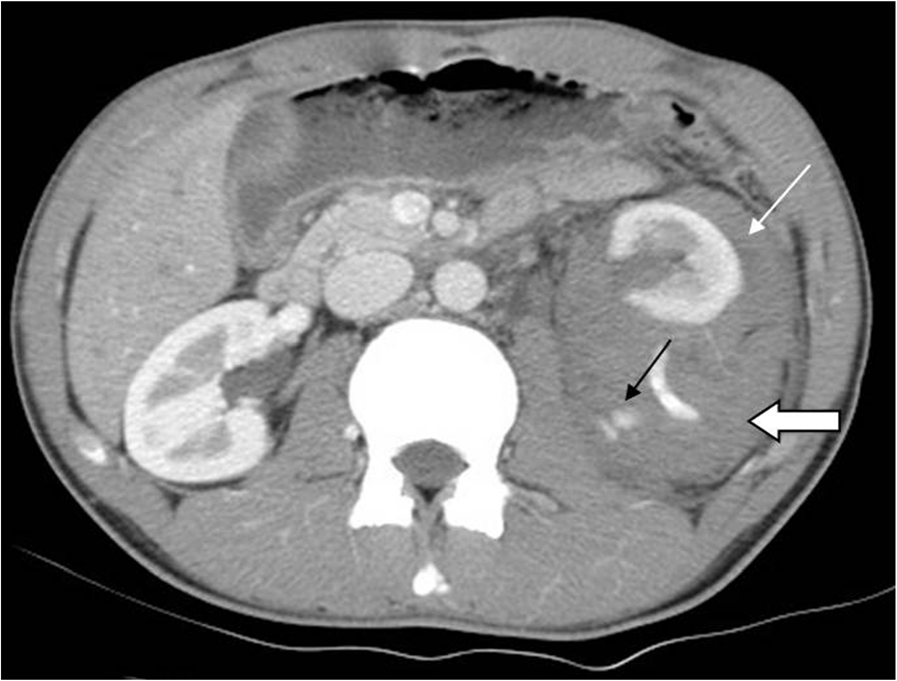
This was a 15 year-old male with grade IV left renal injury from a motor accident. Contrast enhanced CT in arterial phase in axial section showed contrast medium extravasation (*black arrow*), perirenal hematoma (*open arrow*) and pararenal hematoma (*white arrow*)

**Fig. 2:**
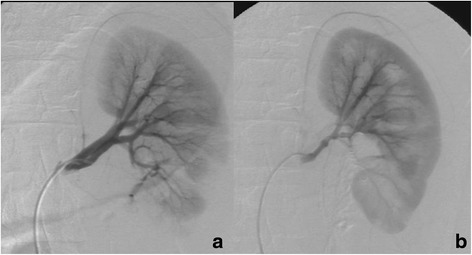
**a**,**b** A 15-year-old young male with grade IV renal injury, whose angiography of left renal artery revealed active contrast medium extravasation at low pole of left kidney (**a**). After superselective embolization of the renal artery by stainless steel microcoils via 3 Fr microcatheter, immediate renal angiography showed no evidence of contrast medium extravasation or other abnormal vascular stain (**b**)

Six patients underwent angiography because CT demonstrated active hemorrhage (Fig. 1), and 4 of them required TAE because of persistent extravasation of the contrast medium found during angiography (Fig. 2). In these four older children (14–16 years old), a microcatheter was used in only one patient, and the other 3 patients with proximal segmental arterial injury underwent superselective TAE without the use of a microcatheter. The two patients who did not receive TAE also did not require subsequent nephrectomy.

Blood transfusion in 24 h was required in 11 patients (61 %) with the average amount of blood transfused being 3.0 ± 3.0 units. Of these, three patients (75 %) were from the TAE group, and 8 (57 %) from the non-TAE group. No patients needed blood transfusion in the non-TAE group after 24 h, while two patients required blood transfusion following TAE or angiogram group after 24 h: one patient (number 3) required 4u blood transfusion because he underwent operation for epidural hemorrhage and intracranial hemorrhage, and another one (number 6) required 2u. Serum creatinine was normal (<1.5 mg/dL) in all patients at the time it was measured in the emergency room. Hematuria was detected in three patients (75 %) in the TAE-group and 12 patients (86 %) in the non-TAE group.

There were no significant differences between the TAE group and non-TAE group with regard to patient demographics (Table 2).

**Table 2:** Comparison of TAE group and Non-TAE group

	TAE group (*n* = 4)	Non-TAE group (*n* = 14)	*p* value
Age	15 ± 0.82	13.64 ± 4.85	0.333
Grade	3.75 ± 0.96	3.29 ± 1.14	0.756
Hematuria	3/4	12/14	0.456
ISS score	19.75 ± 8.99	17.5 ± 8.11	0.638
Shock index	0.72 ± 0.34	0.89 ± 0.30	0.347
Hemoglobin	13.78 ± 1.43	12.99 ± 1.86	0.452
Creatinine	0.92 ± 0.20	0.86 ± 0.23	0.659
Patient number of blood transfusions	3 (75 %)	8 (57 %)	0.151
Blood transfusion amount (u)	4.0 ± 3.27	2.5 ± 3.0	0.400
Hospital stay (days)	11.0 ± 5.48	9.9 ± 11.33	0.850

*ISS* injury severity score, *TAE* transcatheter angiographic embolization

In the non -TAE group, all were treated with conservative medical management except one (number 7) required surgery, who underwent a nephrectomy with simultaneous splenectomy due to persistent renal arterial hypertension and associated active splenic hemorrhage; global renal infarction was found during the laparotomy. This patient’s hypertension resolved after nephrectomy.

Procedure-related complications included post embolization hypertension, contrast-induced nephropathy (with angiography and TAE) were not seen. All patients survived with normal renal function, and no patients had a recurrence of renal bleeding at the 6-month follow-up.

## Discussion

Although TAE is an established treatment modality in adults used commonly for blunt renal injury for active renal hemorrhage, with high rates of renal salvage and renal function preservation; it is rarely used in children because of the smaller size of pediatric arteries and associated concern about procedure-related safety and complications [10–12]. Vast majority of interventional radiologists do not have exceptional experience or comfort level in treating children, thus a rate limiting access issue. This leads to comfort of TAE in children to be referred by clinical trauma services. We report our experience with interventional radiology for pediatric blunt renal injury, in which there were no cases of complications after TAE.

There are some differences of blunt renal injury between children and that in adults. For one, hypotension is relatively rare in children compared to adults and does not appear to be a clinically useful indicator in management of pediatric blunt renal injury because it is a late sign of impending hemodynamic collapse [14]. Tachycardia, an important earlier sign of volume or blood loss, should be observed closely in children with blunt renal injury before their blood pressure drop. Another important difference is that blood vessels with enhanced vasoconstrictive response is smaller in children; result in bleeding associated with solid viscus injury usually can be successfully stopped without surgery. In this study, no patient had hypotension; but one of the three patients without hematuria had significant grade 4 renal injury and later received TAE.

Imaging guidelines for pediatric blunt renal trauma have been proposed but there has been no consensus. Stein et al. proposed that all children with renal trauma having any level of hematuria should undergo an abdominal CT scan [15]; however, in a recent study, Nguyen et al. recommended that the decision for renal imaging for the diagnosis and grading of pediatric renal injuries should not be only depended on urinalysis without considering the clinical condition, history, and causes of injury [1]. Therefore, physical examination or laboratory results suggestive of an abdominal injury including hematuria, abdominal bruising or ecchymosis, abdominal pain/distention, absent bowel sound, vomiting, low hematocrit level, and blood from the rectum or nasopharyngeal tube aspirate in children with blunt abdominal trauma are all indications for an imaging study.

Ultrasonography is recommended for initial screening and follow-up, but its actual diagnostic outcome for different grades of renal injury is quite dismal; moreover, its accuracy depends on the performer’s experience [16]. CT scanning is the gold standard radiographic assessment for stable patients with blunt renal injuries, as it can diagnose the grade of hemorrhage from the kidney, morphological changes of the kidney, the presence of urinoma [15–17], and can also simultaneously evaluate other organ injuries [1, 17]. This study revealed 3 patients without hematuria who had grades 2, 3, and 4 renal trauma, respectively, proven by CT scan later.

Non-operative management of blunt renal trauma is progressively acceptable by clinical physicians, demonstrating effectiveness in more than 85 % of patients [12, 18]. Surgical intervention is reserved for patients with hemodynamic instability, renal venous pedicle injury, and failure of conservative medical treatment. It has been shown that even among patients with grade 5 renal trauma, up to 80 % can be treated successfully by nonoperative management in the absence of hemodynamic instability [8, 19, 20].

Angiography and selective TAE are generally reserved for patients in hemodynamically stable or marginally unstable conditions with active hemorrhage detected on CT scan or delayed hemorrhage that occurs when the patient is under nonoperative management. The indications of TAE for renal injury are as follows: i.) signs suggestive of vascular renal injury, including parenchyma laceration, contrast extravasation, and perirenal hematoma seen by CT, ii.) arteriovenous fistula, or pseudoaneurysm with evidence of persistent bleeding [10, 21, 22]. There have been very few reports in literature of TAE being used in pediatric renal trauma. Dinkel et al. have shown the effectiveness and safety of superselective TAE in 9 severe blunt renal trauma patients, of which 3 had grade 5 blunt renal trauma [23]. Eassa et al. reported two children with grade 5 injury who underwent TAE, and recommended that TAE could be an excellent option for grade 5 renal injury in children with continuing hemorrhage and who have pseudoaneurysms [12]. Vo NJ et al. reported 7 pediatric renal trauma patients who required embolization, and concluded that TAE is relatively safe and potentially effective in the setting of abdominal and pelvic trauma in the pediatric population [24].

Radiation exposure is an important concern for pediatric patients. All our CT scanners are multi-detector CT, and they have automatic exposure control devices to decrease the radiation dose. CT dose reduction in pediatric imaging requires a combination of different approaches or strategies; we will work on optimization of scanning protocols for children according to age-or weight-based adjustments, decreasing the unnecessary examination and scanning phase, and user education for pediatricians and radiological technologies.

The complications of TAE in children are similar to those in adults, but incidence of complications is relatively lower in children than in adults [12]. These include arterial puncture site hematoma, catheter- or guidewire-related arterial injury, contrast-induced nephropathy, target organ ischemia, and nontarget organ embolization (13). In this study, TAE successfully stopped renal bleeding in 4 high-grade renal injury patients without procedure-related complications.

The complications of renal injuries are urinary tract infection, recurrent gross hematuria, obstructive uropathy related to blood clot, urinoma, and hypertension [4, 13]. The reported incidence of posttraumatic hypertension ranges from 0–15 % in pediatric literature [2, 8, 25, 26]. Hypertension may result from ischemic renal tissue, renal artery thrombosis, devitalized fragments and arteriovenous fistulae. Treatment such as medical management, excision of the ischemic segment or total nephrectomy is required in patients with persistent hypertension [27, 28]. In this study, one (7 %) patient in the non-TAE group developed persistent renal vascular hypertension directly related to trauma which was cured after a nephrectomy.

There are some limitations to this study. The most important one is that the sample size was small. In addition, the records of children with blunt renal trauma were collected retrospectively, which may have resulted in patient selection bias, and overuse of TAE may attract criticism; however, while a prospective study would have thoroughly perfomed the benefit of this approach in stable patients, it might have been impossible in the setting of acute trauma care. Also, TAE was performed based on positive angiography findings similar to those reported in many previous studies in adults; we tried to apply the same indications and reappraise its role in children [19, 21, 23].

## Conclusion

When positive vascular findings of contrast medium extravasations are found on the angiography in children with blunt renal injury, our results suggest that intervention of TAE is indicated, which is a useful treatment option to minimize renal parenchyma damage. This study was based only on the experience of one hospital; further multiple medical centers’ studies should focus on redefining indications and comforting level of interventional radiologists for TAE in pediatric blunt renal trauma patients.
